# Central Role of CD169^+^ Lymph Node Resident Macrophages in the Adjuvanticity of the QS-21 Component of AS01

**DOI:** 10.1038/srep39475

**Published:** 2016-12-20

**Authors:** Sophie Detienne, Iain Welsby, Catherine Collignon, Sandrine Wouters, Margherita Coccia, Sophie Delhaye, Laurye Van Maele, Séverine Thomas, Maëlle Swertvaegher, Aurélie Detavernier, Abdelatif Elouahabi, Stanislas Goriely, Arnaud M. Didierlaurent

**Affiliations:** 1 Institute for Medical Immunology (IMI), Université Libre de Bruxelles, Rue Adrienne Bolland 8, B-6041 Gosselies, Belgium; 2 GSK Vaccines, Rue de l’Institut 89, B-1330 Rixensart, Belgium

## Abstract

Saponins represent a promising class of vaccine adjuvant. Together with the TLR4-ligand MPL, QS-21 is part of the Adjuvant System AS01, a key component of the malaria and zoster candidate vaccines that display demonstrated clinical efficacy. However, the mechanism of action of QS-21 in this liposomal formulation is poorly understood. Upon intra-muscular immunisation, we observed that QS-21 rapidly accumulated in CD169^+^ resident macrophages of the draining lymph node where it elicited a local innate immune response. Depletion of these cells abrogated QS-21-mediated innate cell recruitment to the lymph node, dendritic cell (DC) phenotypic maturation as well as the adjuvant effect on T-cell and antibody responses to co-administered antigens. DCs rather than lymph node-resident macrophages were directly involved in T-cell priming by QS-21, as revealed by the decrease in antigen-specific T-cell response in *Batf3*^−/−^ mice. Further analysis showed that the adjuvant effect of QS-21 depended on the integration of Caspase-1 and MyD88 pathways, at least in part through the local release of HMGB1. Taken together, this work unravels the key role of lymph node sentinel macrophage in controlling the adjuvant effect of a molecule proven to improve vaccine response in humans.

Purified recombinant/sub-unit antigens elicit modest antibody responses. Therefore, vaccines containing these antigens also include immunostimulatory molecules known as adjuvants, that can enhance and shape specific immune responses^1^. These immunostimulatory molecules are thought to activate innate immune cells acting either as pathogen-associated molecular patterns (PAMPs) or by inducing danger-associated molecular patterns (DAMPs).

Among the many adjuvants tested in humans so far, the Adjuvant System AS01 is the only one able to induce protective immunity against malaria and is a key component of the zoster vaccine that recently showed a 97% efficacy in the elderly^2^,^3^,^4^. AS01 is a liposome-based adjuvant comprising 3-*O*-desacyl-4′-monophosphoryl lipid A (MPL), a Toll-like receptor 4 ligand and QS-21, a saponin extracted from the bark of the *Quillaja saponaria* Molina tree. The adjuvant has been shown to induce robust antigen-specific cellular and humoral adaptive responses (reviewed in Garçon *et al*.^5^). Furthermore, recent data obtained in mouse models have shown that AS01 induces a rapid activation of the innate immune response both at the site of injection and in the draining lymph node. Immunisation with AS01-adjuvanted recombinant proteins leads to the recruitment of antigen-loaded dendritic cells to the draining lymph node that is critical for the induction of antigen-specific adaptive responses^6^.

While AS01 induces potent immune cellular and humoral responses, the exact role of the QS-21 component is poorly characterised. Saponins exhibit a diverse range of biological activities such as immunomodulatory, anti-tumoral and anti-microbial properties^7^. QS-21 is a water soluble triterpene glycoside with amphiphilic character and was found to possess a high level of adjuvant activity combined with a comparatively low level of toxicity^8^. However, it still possesses haemolytic activity that can be eliminated through adequate formulation^9^. Two structural features of QS-21 have been identified as being critical for its adjuvant activity: the triterpene aldehyde and the fatty acyl side chain^10^. QS-21 has been shown to enhance CD8 T-cell responses in mice, possibly through the promotion of antigen cross-presentation by dendritic cells, as has been described for other saponins^11^,^12^. Moreover, QS-21 promotes the production of antigen-specific antibodies both in mice^13^ and in humans^7^,^14^,^15^. Whether QS-21 activates cells via a specific receptor remains unknown, and the signalling pathways it induces are also still poorly understood. QS-21 has been shown to activate the inflammasome *in vitro*^16^. Furthermore, a recent report has demonstrated that QS-21 activates ASC-NLRP3 inflammasome and subsequent IL-1β/IL-18 release^17^. Finally, ISCOMATRIX, an adjuvant containing saponins, also activates the inflammasome *in vitro*, which can lead to IL-18 release *in vivo* that is important for CD8 T cells and IgG2c antibody responses^18^.

The goal of this study was to identify mechanisms involved in the activation of the immune system by the QS-21 component of AS01. We identified CD169^+^ resident macrophages of the lymph node draining the injection site as the main cells targeted by QS-21 when formulated into liposomes.

## Results

### QS-21 incorporated in liposomes induces robust adaptive immune responses to co-administered antigens

**Figure 1:**
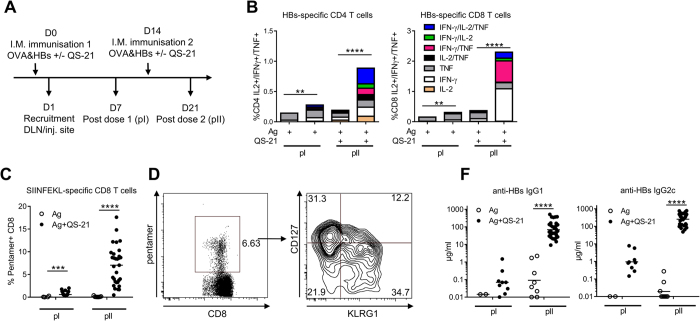
QS-21 is a potent adjuvant for the induction of cellular and humoral responses. (**A**) Experiment timeline. (**B**) Mice were immunised as in (**A**) and cytokine production and polyfunctionality of HBs-specific CD4 and CD8 T cells were assessed by flow cytometric cytokine intracellular staining at days 7 and 21. Data is represented as median of 6 (Ag alone groups) or 20 (Ag + QS-21 groups) animals. (**C**) Percentage of OVA-specific CD8 T cells quantified by H2Kb-SIINFEKL pentamer staining at days 7 and 21. (**D**) Expression of CD127 and KLRG1 on pentamer-positive CD8 T cells measured by flow cytometry. (**F**) Anti-HBs IgG1 and IgG2c titres measured by ELISA in the serum of mice immunised with antigens alone (Ag) or antigens formulated with QS-21 on days 7 and 21. Each point represents a single mouse and the horizontal bar represents the geometric mean. Statistical significance was determined by a non-parametric Mann Mann-Whitney test. The data is representative of at least 2 independent experiments.

QS-21 was incorporated into cholesterol-containing liposomes, similarly to the clinical AS01 formulation^9^. To assess the adjuvanticity of this QS-21 formulation, mice were immunised intramuscularly against two model antigens (Ag): HBs, a clinically relevant Ag used in the *Engerix*™ vaccine, and OVA that allows tracking of Ag-specific CD8 T cells using SIINFEKL-H2Kb pentamers following the protocol described in Fig. 1A. Ag-specific responses were measured either 7 days after the first (post dose I) or second (post dose II) injection. Upon restimulation with HBs peptides *in vitro*, the frequency of cytokine-producing CD4 and CD8 T cells was low post dose I, but increased significantly post dose II in the group receiving antigen co-administered with QS-21 as compared to antigen alone. Furthermore, a robust increase in the number of cells producing simultaneously two or three cytokines was observed after the second immunisation (Fig. 1B and Supplementary Fig. S1). Similarly to HBs-specific CD8 T cells, QS-21 strongly supported the expansion of OVA-specific CD8 T-cells, especially post dose II (Fig. 1C). Ag-specific effector CD8 T cells differentiate into two main subsets: terminally differentiated effector (TE) cells that are CD127^−^ and KLRG1^+^ and memory precursor (MP) cells that are CD127^+^ KLRG1^−^ and can become long-lived memory cells^19^. Immunisation with QS-21-adjuvanted Ags led to the differentiation of both TE and MP CD8 T-cell subsets (Fig. 1D). Finally, QS-21-also increased anti-HBs IgG1 and IgG2c production when co-administered with antigens, establishing QS-21 in a liposome formulation as a robust adjuvant inducing both cellular and humoral responses following a prime-boost regimen (Fig. 1F).

### Intramuscular injection of QS-21 leads to a rapid local innate response in the draining lymph node

**Figure 2:**
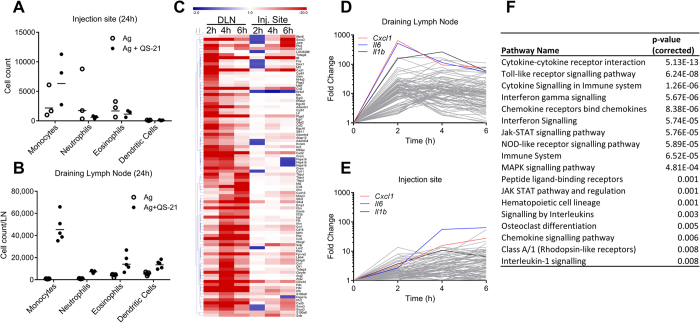
QS-21 induces rapid changes in gene expression in draining lymph node. (**A**,**B**) Monocyte (CD11b^+^ Ly6C^+^ Ly6G^−^), neutrophil (SSC^hi^ CD11b^+^ Ly6C^int^ Ly6G^+^), eosinophil (SSC^hi^ CD11b^+^ Ly6C^+^ Ly6G^−^ SiglecF^+^) and dendritic cell (CD11c^+^ MHCII^+^) recruitment was assessed by flow cytometry 24 h post immunisation in the muscle (**A**) and draining lymph node (**B**). Each point represents a pool of 2 (DLN) or 3 (muscle) mice and the horizontal bar is the geometric mean. (**C**) Heatmap representation of mRNA expression (Fold change over PBS) of the top upregulated genes (Fold Change over PBS >10 in either the injection site or draining lymph node) measured by microarray analysis. (**D**,**E**) Fold change over PBS of the top 50 upregulated genes at 2, 4 or 6 hours in the draining lymph node (**D**) or injection site (**E**). The top upregulated genes in the draining lymph node (*Cxcl1, il6, il1b*) are represented on each chart. (**F**) InnateDB analysis of pathway over-representation of significantly upregulated genes (p-value < 0.05 and fold change >3) using the hypergeometric algorithm and Benjamini Hochberg correction for p-values.

In order to assess local innate responses that may be linked to the adjuvant effect of QS-21, PBS or QS-21 were administered i.m. to mice and innate cell recruitment was measured in both the muscle and the draining lymph node (DLN). Twenty-four hours post-injection, low or no recruitment of monocytes or neutrophils were observed at the injection site (Fig. 2A). In contrast, at the same time point, accumulation of monocytes, neutrophils, eosinophils and dendritic cells was readily detectable in the draining lymph node (Fig. 2B). Increase in dendritic cell number was low (2-fold increase) when compared to monocytes and neutrophils (>10-fold increase). Microarray analysis of gene expression at the injection site and in the DLN was performed 2, 4 or 6 hours after i.m. injection. A heatmap representation of the top upregulated genes (QS-21/PBS fold change >10 in either the DLN or injection site at either 2, 4 or 6 hours) shows that the response was both faster and of higher magnitude in the DLN compared to the injection site (Fig. 2C). The variations over time of expression of the top 50 upregulated genes in either the injection site or DLN were also represented as stacked line charts (Fig. 2D,E). These representations highlight the fact that the magnitude and speed of induction of gene expression is greater in the DLN than at the injection site. Analysis of overrepresented immune signalling pathways in the DLN using the InnateDB web resource^20^ shows that QS-21 induced innate immune activation, with the top pathways involving cytokine, chemokine, TLR or NLR signalling (Fig. 2F).

### QS-21 is drained to the lymph node where it colocalises with CD11b^+^ CD169^+^ resident macrophages

**Figure 3:**
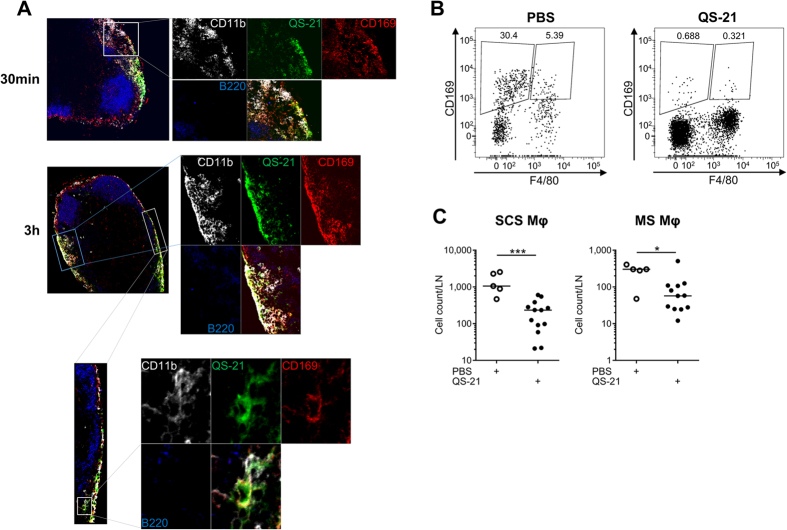
QS-21-bodipy rapidly accumulates in the draining lymph node where it colocalises with CD11b^+^ CD169^+^ macrophages of the subcapsular sinus. (**A**) Mice were injected i.m. with liposomes containing bodipy-labelled QS-21. The draining lymph nodes were recovered 30 min and 3 h post injection and stained with anti-CD11b, anti-CD169 and anti-B220 antibodies and analysed by confocal microscopy. (**B**) Flow cytometry analysis of subcapsular sinus (CD169^+^ F4/80^−^) and medullary sinus (CD169^+^ F4/80^+^) macrophages in mice 24 h following PBS or QS-21 injection. (**C**) Quantification of the loss of LN-resident macrophages detected by flow cytometry. Results from two independent experiments. Each point represents one mouse. Statistical significance was determined by a non-parametric Mann-Whitney test.

The microarray data (Fig. 2C–E) identified a rapid innate activation signature in the DLN. Consistently with this finding, recent results have shown that fluorescently labelled QS-21 incorporated in AS01 is rapidly drained to the subcapsular region of the lymph node^6^. Using a similar experimental setting, the cells targeted by QS-21 *in vivo* were further analysed by confocal microscopy. The draining (iliac) lymph nodes were recovered 30 min and 3 h following injection and visualised by confocal microscopy. Bodipy-labelled QS-21 accumulated in the subcapsular sinus (SCS) as early as 30 min following injection (Fig. 3A) and colocalised at both time points with CD11b^+^ CD169^+^ cells, which are macrophages known for their ability to capture lymph-borne particles^21^. As previously demonstrated in the context of lymph-borne infections^22^,^23^, immunisation with QS-21 led to a rapid loss of lymph node macrophages, the CD11b^+^ CD169^+^ F4/80^−^ SCS subpopulation being more affected than CD11b^+^ CD169^+^ F4/80^+^ medullary sinus macrophages (Fig. 3B,C).

### Lymph Node Resident macrophages are critical for innate and effector responses to antigens adjuvanted with QS-21

**Figure 4:**
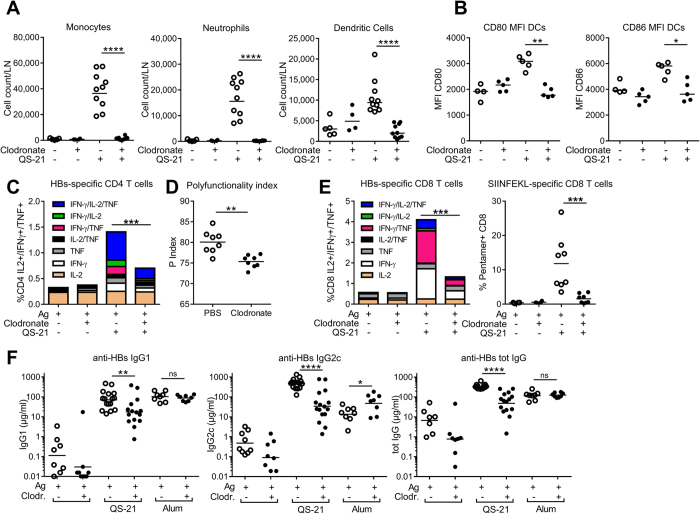
Clodronate-mediated depletion of CD169^+^ subcapsular sinus macrophages greatly reduces innate and effector responses induced by QS-21. (**A**) Monocyte, neutrophil and dendritic cell recruitment to the DLN measured by flow cytometry 24 h post QS-21 injection (Ag: n = 5, QS-21: n = 10). (**B**) Expression of the co-stimulatory molecules CD80 and CD86 on dendritic cells measured by flow cytometry 24 h post QS-21 injection (Ag: n = 4, QS-21: n = 5) (**C**) Mice were injected with clodronate or control liposomes 6 days prior to vaccination and immunised as in 1 A. At day 21, median cytokine production (**C**) and polyfunctionality (**D**) of HBs-specific CD4 T cells, cytokine production by HBs-specific CD8 T cells (**E**), and frequency of OVA-specific circulating CD8 T-cells (**E**) were assessed by flow cytometry (Ag: n = 4, QS-21: n = 8). (**F**) Anti-HBs IgG1, IgG2c and total IgG titres in the serum of mice injected with QS-21 or Alum were measured by ELISA at day 21 (Ag: n = 8, QS-21: n = 16, Alum: n = 8). Each point represents a single mouse and the horizontal bar represents the geometric mean. Statistical significance was determined by a non-parametric Mann-Whitney test. The data is representative of at least 2 independent experiments.

LN-resident macrophages have been shown to initiate the response to and limit the dissemination of various pathogens and particles^24^,^25^,^26^. To determine the role of resident macrophages in the response to QS-21, these cells were depleted by i.m. injection of clodronate-containing liposomes (CL)^27^ 6 days prior to the first immunisation. Mice were immunised following the protocol described in Fig. 1A. Six days post CL injection, depletion was limited to CD169^+^ macrophages of the DLN, with no significant effect on other innate cell populations (Supplementary Fig. S2). Macrophage depletion resulted in the abrogation of monocyte, neutrophil and dendritic cell recruitment to the DLN 24 h post-immunisation (Fig. 4A). Clodronate treatment suppressed QS-21-induced upregulation of CD80 and CD86 by dendritic cells (DCs), indicating that macrophages are required for their phenotypic maturation (Fig. 4B). The adaptive response was also affected by CL-treatment, as depletion of LN-macrophages led to a strong decrease in the frequency of HBs-specific CD4 (Fig. 4C) and HBs- and OVA-specific CD8 (Fig. 4E) T cells. Polyfunctional T cells (i.e. cells simultaneously producing a combination of effector cytokines) can be crucial determinants for the efficacy of vaccines^28^. CD4 T-cell polyfunctionality was quantified following a previously described index^29^. Remaining CD4 T cells from CL-treated animals showed decreased polyfunctionality when compared to controls (Fig. 4D). Finally, the HBs-specific IgG1, IgG2c and total IgG titres were strongly decreased after CL-mediated macrophage depletion, with little effect on the antibody responses induced by alum-adjuvanted antigens, indicating that the effects of macrophage depletion are most likely specific to QS-21 (Fig. 4F).

### Batf3-dependent dendritic cells regulate QS-21-mediated CD4 and CD8 T-cell responses

**Figure 5:**
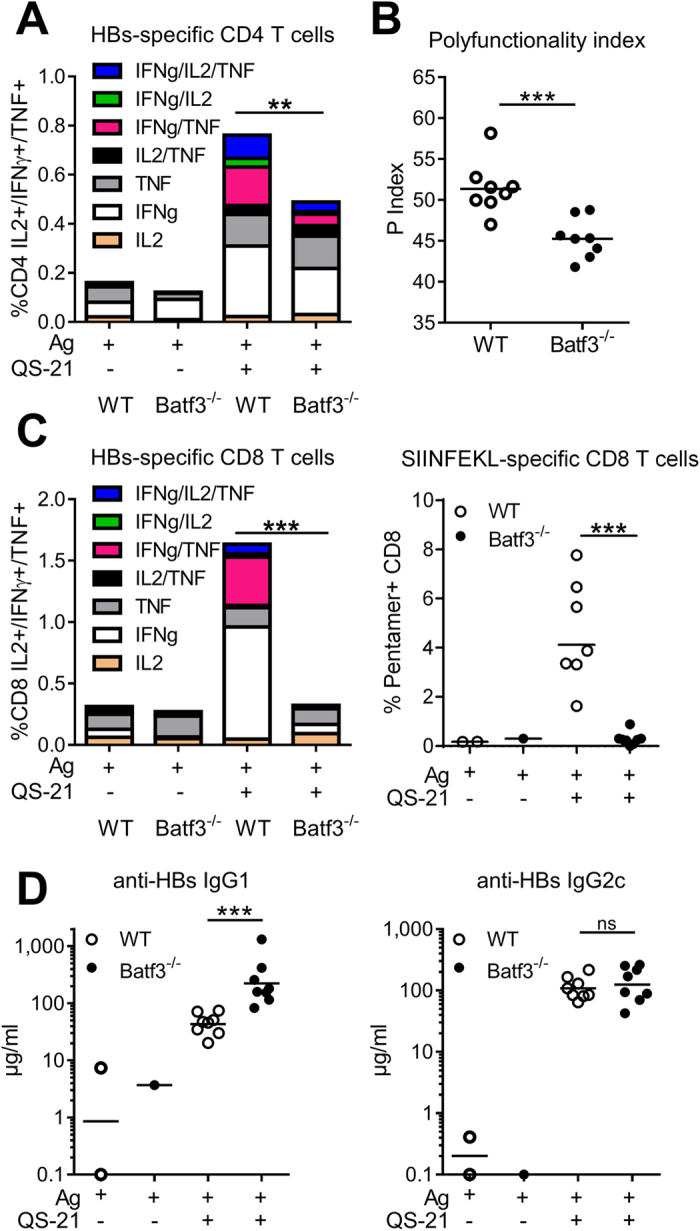
Batf3-dependent dendritic cells regulate QS-21-mediated T-cell responses. Mice were immunised as in 1 A and adaptive responses were measured at day 21. (**A**) Flow cytometry analysis of cytokine production by HBs-specific CD4 T cells in WT and *Batf3*^−/−^ mice. (**B**) polyfunctionality index of CD4 T cells. (**C**) Flow cytometry analysis of cytokine production by splenic HBs-specific CD8 T cells and of the frequency of circulating OVA-specific CD8 T cells (Ag: n = 1–2, QS-21: n = 8). (**D**) Anti-HBs IgG1 and IgG2c titres in the serum measured by ELISA (Ag: n = 1–2, QS-21: n = 8). Each point represents one mouse and the horizontal bar represents the geometric mean. Statistical significance was determined by a non-parametric Mann-Whitney test.

Although SCS macrophages may directly present peptide antigens to effector T cells^30^, other antigen-presenting cells could be involved in direct antigen presentation. Dendritic cells are the most potent inducers of T-cell proliferation, and their recruitment and activation were decreased following CD169^+^ macrophage depletion. In order to determine the potential involvement of specific DC subsets in QS-21-induced Ag-specific T-cell responses, mice lacking expression of BATF3 were immunised as in Fig. 1A. This model was favoured, as classical DC depletion models can also deplete macrophage subpopulations that express CD11c^31^. *Batf3*^−/−^ mice display severe defects in the development of CD8α^+^ and CD103^+^ dendritic cells^32^,^33^, subsets that mediate antigen cross-presentation. No CD103^+^ dendritic cells were detectable in the DLN of BATF3-deficient mice, whereas CD169^+^ cell numbers in the DLN were comparable to WT mice (Supplementary Fig. S3). Upon immunisation, the frequency and polyfunctionality of HBs-specific CD4 T-cells responses were significantly reduced in BATF3-deficient animals (Fig. 5A,B) while both OVA- and HBS-specific CD8 T-cell responses were completely abolished (Fig. 5C). However, the altered CD4 T-cell responses did not translate into differences in antibody levels, with increased IgG1 and normal IgG2 titres in *Batf3*^−/−^ mice (Fig. 5D).

### Inflammasome activation and Caspase 1/11-dependency of the innate and effector responses to QS-21

**Figure 6:**
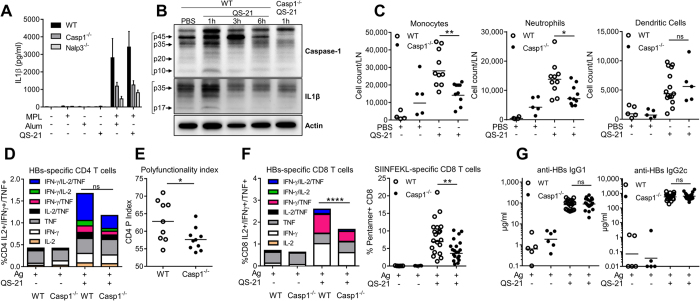
QS-21 elicited innate cell recruitment and CD4/CD8 T-cell responses are dependent on Caspase-1. (**A**) Bone-marrow derived dendritic cells (BMDCs) from mice lacking NALP3 or Caspase-1 expression were primed overnight with MPL and stimulated with either QS-21 or alum. IL-1β release in the supernatant was determined by ELISA. The data is represented as mean and SEM of 3 independent experiments. (**B**) WT or caspase-1 KO mice were injected *i.m.* with either PBS or QS-21 and the draining lymph nodes were recovered at the indicated time points. Whole lymph nodes were lysed and caspase-1 and IL-1β processing were detected by western blot. (**C**) Innate cell recruitment to the iliac DLN in WT and Caspase-1 KO mice 24 h post QS-21 injection. Absolute numbers of monocytes (Lin-CD11b^+^ Ly6C^+^ Ly6G^−^), neutrophils (SSC^hi^ Lin^−^ CD11b^+^ Ly6C^int^ Ly6G^+^) and dendritic cells (Lin^−^ CD11c^+^ MHCII^+^) per DLN were assessed by flow cytometry (PBS: n = 5, QS-21: n = 5–10). (**D**–**F**) Mice were immunised as in 1 A. At day 21, cytokine production (**D**) and polyfunctionality (**E**) of HBs-specific CD4 were evaluated by flow cytometry. (**F**) Cytokine production of HBs-specific and frequency of OVA-specifc CD8 T cells were evaluated by flow cytometry (Ag: n = 3, QS-21: n = 9–10). (**G**) Anti-HBs IgG1 and IgG2c titres in the serum at day 21 were measured by ELISA (Ag: n = 5–6, QS-21: n = 20). Each point represents one mouse and the horizontal bar represents the geometric mean. Statistical significance was determined by a non-parametric Mann-Whitney test. The data is representative of 2 independent experiments.

Next, we sought to determine the early innate signalling pathways that are triggered locally by QS-21. Several QuilA derivatives were previously shown to activate the inflammasome^16^,^17^,^18^. To assess the capacity of QS-21 to activate the canonical inflammasome pathway, bone marrow-derived dendritic cells from wild-type mice or mice invalidated for NALP3 or Caspase-1 were either directly stimulated with MPL, alum or QS-21 or primed with MPL followed by stimulation with alum or QS-21. In this model, priming is required for optimal inflammasome activation and secretion of IL-1β^34^. Figure 6A shows that IL-1β levels induced by QS-21 were similar to alum, and dependent on Caspase-1 and NALP3, indicating classical inflammasome activation. Moreover, QS-21 injection in the gastrocnemius muscle led to detectable caspase-1 activation and IL-1β processing in the DLN 1 h post-injection, as shown by the detection of the p10 and p17 subunits of caspase-1 and IL-1β, respectively (Fig. 6B).

We then examined the influence of Caspase-1/11 deficiency on the adjuvant effect of QS-21 following the protocol described in Fig. 1A. Twenty four hours post-immunisation using QS-21, monocyte and neutrophil recruitment to the draining lymph node was significantly reduced, while dendritic cell numbers were not decreased (Fig. 6C). Seven days after the second immunisation, *ex vivo* restimulation of spleen cells with antigenic peptides showed no significant decrease in cytokine production by HBs-specific CD4 T cells (Fig. 6D). However, the quality of the response was altered as CD4 T-cell polyfunctionality was reduced in Caspase-1/11 deficient animals (Fig. 6E). Similarly, the frequencies of cytokine-producing HBs-specific CD8 T cells and of circulating OVA-specific CD8 T cells were significantly reduced (Fig. 6F). However, despite these effects on effector and innate responses, HBs-specific IgG2c and IgG1 titres elicited by QS-21 formulation in caspase-1/11 knockout mice were comparable to their wild-type counterparts (Fig. 6G). These observations indicate that Caspase-1 is involved in QS-21-induced innate cell recruitment and both CD4 and CD8 T-cell responses but not antibody responses.

### MyD88-mediated signalling events are required for optimal responses to QS-21

**Figure 7:**
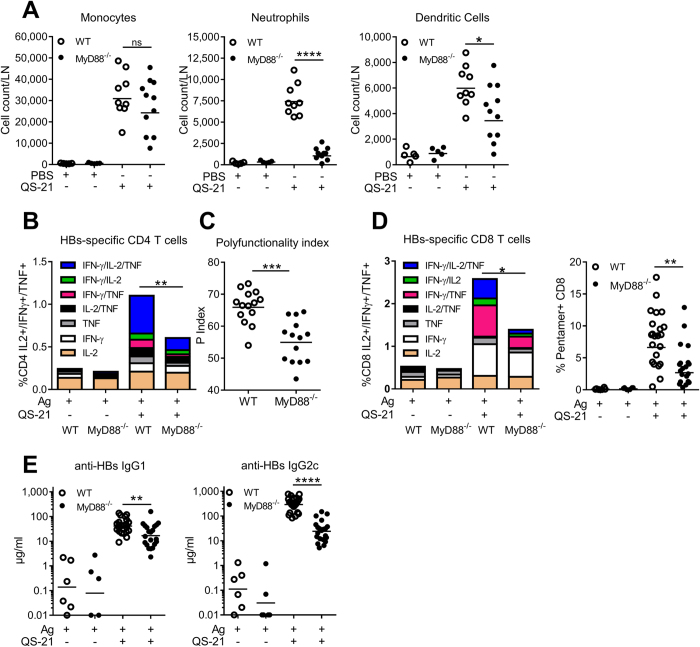
QS-21-elicited neutrophil and dendritic cell recruitment, cellular CD4/CD8 T-cell responses and antibody production are dependent on MyD88. (**A**) Innate cell recruitment in the iliac DLN 24 h post QS-21 injection. Absolute numbers of monocytes (Lin^−^ CD11b^+^ Ly6C^+^ Ly6G^−^), neutrophils (SSC^hi^ Lin^−^ CD11b^+^ Ly6C^int^ Ly6G^+^) and dendritic cells (Lin^−^ CD11c^+^ MHCII^+^) per DLN were assessed by flow cytometry (PBS: n = 6, QS-21: n = 7–8). (**B**–**D**) WT and MyD88 KO mice were immunised as in 1 A. At day 21, median cytokine production (**B**) and polyfunctionality (**C**) of HBs-specific CD4 were evaluated by intracellular staining (Ag: n = 3, QS-21: n = 14). (**D**) Cytokine production of HBs-specific splenic CD8 T cells and frequency of OVA-specific circulating CD8 T cells assessed by flow cytometry (Ag: n = 3–6, QS-21: n = 14–21). (**E**) Anti-HBs IgG1 and IgG2c titres in the serum at day 21 were measured by ELISA (Ag: n = 6, QS-21: n = 21). Each point represents one mouse and the horizontal bar represents the geometric mean. Statistical significance was determined by a non-parametric Mann-Whitney test. The data is representative of 2 independent experiments.

Caspase-1 activation can lead to the release of IL-1β and IL-18 which signal via their respective receptors, IL-1R and IL-18R. Since MyD88 is a critical adaptor for the signalling downstream of these receptors and of Toll-Like Receptors (TLRs), we evaluated the role of MyD88 in the adjuvant effect of QS-21. WT or MyD88-invalidated mice were immunised according to the protocol described in Fig. 1A. In contrast to the results obtained in Caspase-1-KO animals, MyD88 was dispensable for monocyte recruitment to the DLN. However, neutrophil and dendritic cell recruitment were largely MyD88-dependent (Fig. 7A). The frequency of QS-21-adjuvanted HBs/OVA vaccine-induced cytokine-producing Ag-specific CD4 and CD8 T cells was significantly reduced in MyD88 knock-out mice, as were both the polyfunctionality of CD4 T cells and the proportion of circulating Ag-specific CD8 T cells (Fig. 7B–D). Furthermore, in contrast to the results from Caspase-1/11-deficient mice, HBs-specific IgG2c and IgG1 titres were consistently reduced in MyD88 KO mice (Fig. 7E).

### QS-21-mediated HMGB1 release in the DLN is required for optimal CD4 T-cell responses

**Figure 8:**
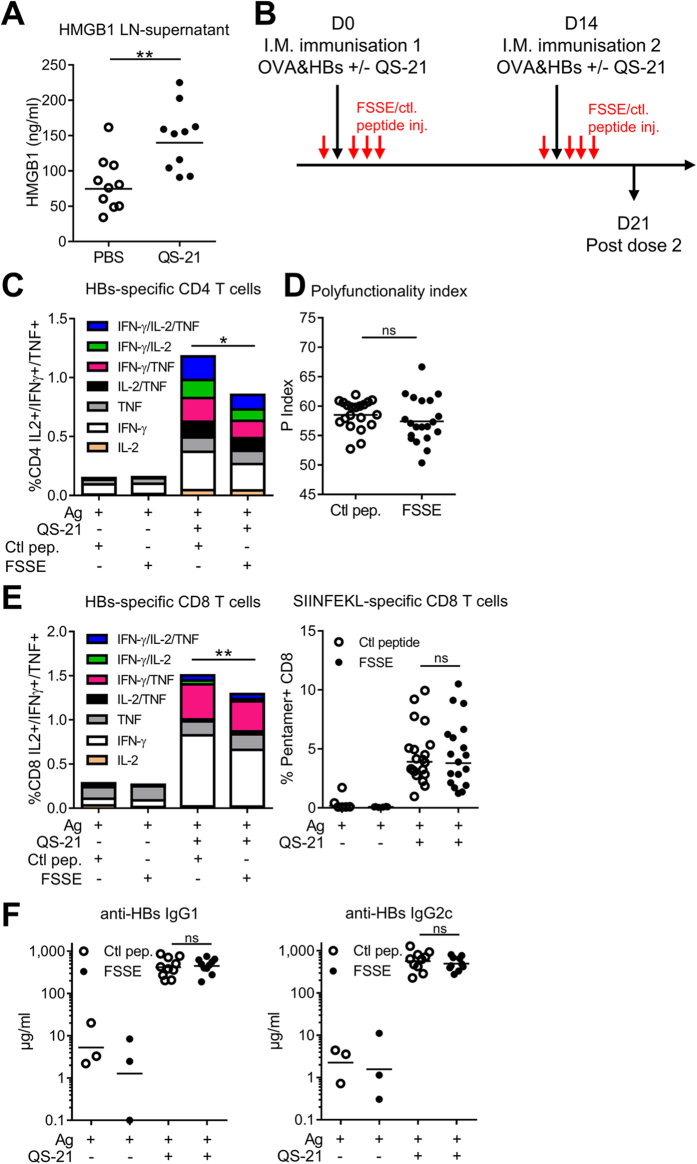
QS-21 injection leads to HMGB1 release that is required for optimal CD4 T-cell responses. (**A**) Mice were injected *i.m.* with QS-21 and the draining lymph nodes were recovered 6 h post injection. The whole lymph nodes were cultured for 24 h in complete medium and HMGB1 was detected in the supernatant by ELISA (n = 10). (**B**) Experiment timeline. Mice received four i.p. peptide (500 μg per mouse – red arrows) injections at day 0 and day 14 (1 h before immunisation and then at 12 h intervals). The FSSE-NH_2_ peptide inhibits HMGB1-MD-2 interaction while the scrambled control (SFSE-NH_2_) does not. (**C**–**F**) FSSE and control peptide-treated mice were immunised as in (**B**). At day 21, cytokine production (**C**) and polyfunctionality (**D**) of HBs-specific CD4 T cells were evaluated after *in vitro* restimulation by intracellular staining. The data is represented as the median of 6 (Ag) or 20 (QS-21) mice. (**E**) Cytokine production by HBs-specific splenic CD8 and frequency of antigen (OVA)-specific circulating CD8 T cells assessed by flow cytometry (Ag: n = 6, QS-21: n = 20). (**F**) Anti-HBs IgG1 and IgG2c titres in the serum at day 21 were measured by ELISA (Ag: n = 3, QS-21: n = 10). Each point represents a single mouse and the horizontal bar represents the geometric mean. Statistical significance was determined by a non-parametric Mann-Whitney test. The data represents a pool of 2 independent experiments.

Since the adaptive responses induced *in vivo* by QS-21 were found to be decreased in caspase1/11- and MyD88-deficient animals, we hypothesised that IL-1 and/or IL-18 signalling events may be involved. We therefore evaluated Ag-specific immune responses elicited upon immunisation of IL-18 and IL-1R-deficient mice. There was no decrease in Ag-specific CD4 or CD8 T-cell responses in either group (Supplementary Fig. S4). Although T-cell responses were maintained, we observed some minor but significant reductions in HBs-specific IgG titres (Supplementary Fig. S5). These results indicate that other mediators contributed to the adjuvanticity of QS-21. Caspase-1 activation is known to trigger pyroptosis and the release of Damage-Associated Molecular patterns (DAMPs). Among these mediators, high-mobility group protein B1 (HMGB1) activates innate response in a MyD88-dependent manner through TLR4 and RAGE^35^. To determine whether QS-21 immunisation leads to HMGB1 release *in vivo*, mice were injected i.m. with QS-21 and the DLNs were recovered after 6 h. The whole lymph nodes were then cultured for 24 h in complete medium and HMGB1 was quantified in the supernatants by ELISA. Figure 8A shows that injection of the QS-21 adjuvant significantly increased HMGB1 release at the time point studied. To investigate the role of this molecule in the adjuvant effect of QS-21, the interaction between HMGB1 and MD-2 (myeloid differentiation factor 2, the TLR4 co-receptor) was inhibited with a specific tetrameric peptide (FSSE-NH_2_)^36^. Mice treated with the HMGB1-inhibitory peptide (FSSE-NH_2_) as in Fig. 8B presented significantly decreased frequency of HBs-specific cytokine-producing CD4 and CD8 T cells (Fig. 8C and E) but no detectable effect on polyfunctionality, OVA-specific CD8 cells or anti-HBs antibody production when compared to the group injected with a scrambled control peptide (SFSE-NH_2_) (Fig. 8D–F). These data indicate that HMGB1-TLR4 signalling partially contributes to the induction of Ag-specific CD4 and CD8 T-cell responses elicited by QS-21 formulation.

## Discussion

Here, we have defined cellular and molecular mechanisms involved in the adjuvant effect of QS-21 when incorporated into cholesterol-containing liposomes. We found that lymph node resident CD169^+^ macrophages act as the key target cells of QS-21 and control its adjuvant effect. Depletion of these cells with clodronate-containing liposomes greatly diminished QS-21-mediated innate cell recruitment and immune responses to co-administered antigens, demonstrating a previously unappreciated role for these macrophages in response to adjuvants. However, CD169^+^ macrophages were not sufficient to drive T-cell responses, as the absence of BATF3-dependent dendritic cells also diminished these responses. We further show that activation of the Caspase-1/MyD88 pathway in the DLN was crucial for the adjuvant effect of QS-21, at least in part through the release of HMGB1.

Subcapsular sinus macrophages rapidly capture lymph-borne particles such as viruses or bacteria, limiting the spread of pathogens to the afferent lymph and the blood^24^,^26^. These cells can also present antigens to B cells and produce cytokines that induce a rapid local innate immune response^25^,^26^. Data shown here and previously published by our group^6^ have revealed that unlike co-administered labelled antigens fluorescent QS-21 (formulated in AS01) primarily colocalised with SCS macrophages shortly after injection, suggesting that liposome-containing QS-21 is specifically retained by those cells. This is in agreement with previously published data showing that SCS macrophages acquire particulate matter while soluble antigens can freely flow into the lymphoid follicle^24^,^25^,^26^,^37^. The loss of CD169^+^ macrophages upon QS-21 injection is reminiscent of the effect of lymph-borne pathogens on these cells. Inflammasome activation in these cells leading to pyroptotic cell death also initiates local innate immune cell recruitment and subsequent adaptive immunity^23^.

Along this line, our data show that clodronate liposome-mediated depletion of LN-resident macrophages inhibited recruitment of monocytes, neutrophils and DCs to the draining lymph node, possibly through modification of the high endothelial venule function^38^. This would indicate that macrophages were the main cells targeted by QS-21 within the first hours post-injection. These observations mirror those of Kastenmüller and colleagues who revealed that SCS macrophages orchestrate the response to pathogens by inducing an IL-18-dependent IFN-γ response in various innate effector cells^26^. In our system, LN-resident macrophage depletion greatly reduced Ag-specific CD4, CD8 and humoral responses to antigens adjuvanted with QS-21 but not with alum, highlighting the difference between liposomal formulations and other adjuvants. CD169^+^ macrophages can play different roles in the regulation of adaptive immune responses. Lymph node SCS macrophages can transfer particulate antigens directly to B lymphocytes, which then migrate to the T cell zone^24^,^25^. CD169^+^ macrophages can also directly cross-present antigens to CD8 T cells in a dendritic cell-independent manner^30^. These observations rely on the fact that CD169^+^ macrophages can capture antigens, retain them and present them to either T cells or B cells. This does not seem to be the mechanism observed with QS-21 for several reasons. First, as previously noted, while QS-21 was retained in the SCS, a co-formulated antigen was rapidly drained to the medulla^6^. Second, mice lacking the CD8α^+^ and CD103^+^ dendritic cell subsets but not CD169^+^ macrophages do not mount CD8 T-cell responses following immunisation with a QS-21-adjuvanted vaccine and exhibit decreased CD4 T-cell responses. Dendritic cells are therefore responsible for the activation of antigen-specific T cells, as previously shown for AS01^6^. Furthermore, preliminary work using an *ex-vivo* presentation assay (as described in Didierlaurent *et al*.) showed that, indeed, resident DCs and monocyte-derived DCs were able to present antigens to T cells after immunisation with a QS21-adjuvanted vaccine. Macrophages are therefore the QS-21-target cells and most likely trigger the early inflammatory response responsible for APC recruitment and activation, and subsequent antigen-specific T-cell priming. Nevertheless, these LN-resident macrophages were critical for the innate and adaptive responses to QS-21-adjuvanted vaccines.

Subcapsular sinus macrophages constitutively produce and store pro-IL-18 that can be released in an inflammasome-dependent manner after bacterial infection, which leads to IFN-γ production by innate lymphoid cells, and they are also able to produce IL-1β, which leads to neutrophil recruitment to the lymph node^26^. In addition, transient inflammasome activation in SCS macrophages also occurs following local viral infection^23^. This activation of the inflammasome leads to a rapid recruitment of innate cells and broadens the scope of T-cell responses^23^. We observed that QS-21 induced canonical NALP3-dependent IL-1β secretion *in vitro.* Additionally, QS-21 injection in the muscle led to detectable caspase-1 and IL-1β cleavage in the draining lymph node after 1 h, when QS-21 is detected in CD169^+^ macrophages. These observations suggest that transient inflammasome activation occurs rapidly in these cells following immunisation with QS-21.

ISCOMATRIX, an adjuvant containing several QuilA saponin fractions also induces inflammasome activation *in vivo* with subsequent IL-1β and IL-18 production, the latter being required for early innate responses^18^. We observed impaired monocyte and neutrophil recruitment to the DLN in Caspase-1/11 deficient mice. QS-21-mediated neutrophil recruitment was also abolished in mice lacking MyD88, but monocyte recruitment was not altered. Neutrophil recruitment was therefore dependent on Caspase-1 and possibly IL-1β/IL-18 signalling while monocyte recruitment seemed to be partially dependent on Caspase-1 but independent of MyD88. This finding is in agreement with previously published data with whole bacteria^39^,^40^. The role of the inflammasome pathway in adaptive cellular and humoral responses elicited by vaccine adjuvants is controversial. Some groups have exposed a role of NLRP3 in Ag-specific antibody production induced by alum, while others have seen no difference between WT and NLRP3-KO mice^41^. Furthermore, a recent report has shown that NLRP3-KO mice displayed increased Ag-specific cellular and humoral response upon immunisation with free QS-21^17^. The importance of the inflammasome pathway in adaptive responses is difficult to pinpoint, given that caspase-1 has multiple substrates that could have diverging effects on the activation and differentiation of lymphocytes^42^. Using QS-21 formulated in liposomes, we observed that caspase 1 and MyD88 pathways contribute to Ag-specific CD4^+^ and CD8^+^ T-cell responses. Our results indicate that this effect was probably mediated by the local release of HMGB1 rather than by IL-1 or IL-18. MyD88 deficiency led to a strong decrease in Ag-specific IgG1 and IgG2c antibody titres, probably reflecting decreased IL-1/18 and TLR signalling in B cells themselves^43^. However, absence of caspase-1 did not affect the action of QS-21 on Ag-specific antibody levels. This could be due to the opposing contribution of IL-1 and IL-18 to the quality of the humoral response. Indeed, lack of IL-1R resulted in reduced Ag-specific IgG1 titres while absence of IL-18 decreased IgG2c but enhanced IgG1 levels.

Taken together, our results show that QS-21 rapidly accumulates in CD169^+^ macrophages of the draining lymph node where it induces caspase-1 activation and HMGB1 release. These early events then orchestrate the recruitment of innate immune cells and activation of dendritic cells. DCs then presumably trigger MyD88-dependent activation of antigen-specific cellular and humoral responses. These observations demonstrate a novel and critical role of resident LN macrophages in the activity of a clinically approved vaccine adjuvant.

## Methods

### Vaccine formulations

QS-21 (*Quillaja saponaria* Molina, fraction 21; licensed by GSK from Antigenics LLC, a wholly owned subsidiary of Agenus Inc., a Delaware, USA corporation) and Hepatitis B surface (HBs) antigen were provided by GSK Vaccines. For the experiments, QS-21 was formulated in DOPC liposomes containing cholesterol at 100 μg/ml. The term “QS-21” used in the manuscript refers to QS-21 formulated in liposomes. Ovalbumin was obtained from Calbiochem and confirmed to be endotoxin-free. All formulations used in this study were made in GSK Vaccines laboratories.

### Mice

C57BL/6 mice were purchased from Harlan Horst. Caspase-1-deficient (Casp1 KO), Batf3-deficient and IL-18-deficient (IL-18 KO) mice on C57BL/6 background were obtained from The Jackson Laboratory. MyD88-deficient (MyD88 KO) and NLRP3-deficient mice on C57BL/6 background were obtained from Dr S. Akira and Dr J.P. Ting, respectively. IL-1 receptor-deficient mice (IL-1R KO mice) mice on C57BL/6 background were kindly provided by Dr B. Ryffel. These mice were housed and bred in our specific pathogen-free animal facility. All animal studies were approved by the Animal Welfare and Ethics Committee of the BIOPOLE ULB CHARLEROI. All experiments were conducted in accordance with the recommended guidelines and regulations.

### Mice immunisations

i.m. injections, containing 4 µg of HBs or 1 µg of OVA, both with 1 µg of formulated QS-21 were performed on 6-to 12-week-old female or male mice, in both hind limbs, in the gastrocnemius muscles and with a volume of 20 μl/muscle. For SCS macrophage depletion, clodronate or control liposomes (Encapsula NanoSciences) were injected 6 days prior to immunisation.

### HBs-specific antibody response

Anti-HBs Ab concentrations were measured by ELISA, using HBs protein as the coating antigen, goat anti-mouse IgG (GAM; SouthernBiotech or Jackson ImmunoResearch) was used as coating for the standard antibody. The HBs protein or the GAM IgG were pre-coated onto a 96-well ELISA plate at a final concentration of 1 μg/ml and 5 μg/ml, respectively, incubated overnight at 4 °C. After blocking, diluted serum samples and the IgG standard (SouthernBiotech) were incubated for 2 hours at RT. Plates were then incubated with Biotin-conjugated goat anti-mouse IgG (SouthernBiotech or Jackson ImmunoResearch) followed by horseradish peroxidase-conjugated streptavidin for 30 min. The plates were incubated in a solution of tetramethylbenzidine (TMB, Invitrogen). The reaction was stopped with 1 M hydrochloric acid. The plates were read at 450 nm on a microplate reader.

### Ag-specific T-cell response

2 × 10^6^ splenocytes from immunised mice were stimulated *in vitro* in a 96-well microplate with HBs or OVA peptide pools at 1 μg/ml. The HBs peptide pool consists of 15-mer peptides with 11 amino acid overlaps encompassing the whole protein. The OVA peptide pool consists of 17 15-mer peptides selected for H2-Kb epitope content. Anti-CD49d and anti-CD28 Abs at 1 μg/ml (BD Biosciences) were added to the culture, and the cells were incubated 2 h at 37 °C. Brefeldin A (1 μg/ml; BD Biosciences) was then added and cells were further cultured over-night at 37 °C. Cell suspensions were washed, resuspended in 50 μl of PBS with 2% FCS, 2 mM EDTA containing 2% Fc blocking reagent (1/50; 2.4G2; BD Biosciences) and stained with anti-CD4 pacific blue (clone RM 4.-5 BD Biosciences; 1/100 final dilution) and anti-CD8-PerCP (clone 53-6.7 BD Biosciences, 1/100 final dilution). Cells were permeabilised in 200 μl of Cytofix-Cytoperm (BD Biosciences), washed with 1 × Perm Wash solution (BD Biosciences) and resuspended with 50 μl 1x Perm Wash buffer containing anti-IFN-γ- APC (clone XMG1.2 BD Biosciences, 1/100), anti-IL-2-FITC (clone JES6-5H4 BD Biosciences, 1/1000) and anti-TNF-PE (clone MP6-XT22 BD Biosciences, 1/2000). Cells were fixed in 200 μl of 1x CellFix solution (BD biosciences) and samples were acquired on a BD LSRFortessa flow cytometer (BD Biosciences). Analyses were performed using FlowJo software. Polyfunctionality was assessed with Funky Cells Data miner as previously described^29^.

For OVA-specific CD8 T-cell responses, 50 μl of whole blood collected with anti-coagulant (heparin, Sanofi-Aventis) was incubated 10 min with 3 μl of PE-labelled MHCI SIINFEKL specific pentamer (ProImmune). Cells were stained with anti-CD4-FITC (clone H129.19 BD Biosciences), anti-CD19-FITC (clone eBio1D3 eBioscience) and anti-CD8-PerCp (clone 53-6.7 BD Biosciences), plus anti-CD127 PE-Cy7 (clone SB/199 BD Biosciences) and anti-KLRG1-APC (Clone 2F1 BD Biosciences) diluted in 50 μl (1/50 final dilution of all antibodies) of PBS 2% FCS-2 mM EDTA. Red cells were then lysed (BD FACS Lysing Solution), washed twice, fixed in 200 μl of 1x CellFix solution (BD biosciences) and acquired with a BD LSRFortessa flow cytometer (BD Biosciences). Analyses were performed using FlowJo software.

### Microarray analysis

Total RNA was isolated by homogenising pooled DLN in Tripure reagent (1 ml/100 mg tissue; Roche Applied Science) and then extracted with chloroform followed by RNeasy Minikit (Qiagen) according to the manufacturer’s protocol. A DNAse treatment was applied on the RNeasy column to avoid genomic DNA contamination. RNA was concentrated by ethanol precipitation, and quantified by RiboGreen (Life Technologies). 1 μg of each RNA sample was used for target preparation, using a one-cycle cDNA synthesis kit, and hybridised to GeneChip Whole Mouse Genome 430 2.0 arrays (Affymetrix). Data acquisition was performed using GeneChip Operating Software (Affymetrix) and data quality control and normalisation was performed with the R and Bioconductor stats packages. Fold changes (QS-21 vs PBS) were calculated for each organ at each time point. A heatmap of the top significantly upregulated genes (FC vs PBS >10, p-value <0.05) was generated with MeV software. The histograms of the top 50 upregulated genes were generated with Graphpad Prism software. Pathway over-representation of significantly upregulated genes (p-value < 0.05 and fold change >3) was performed with the InnateDB web resource using the hypergeometric algorithm and Benjamini-Hochberg correction for p-values^20^. The microarray data are available on the GEO repository with the accession number GSE90864

### SDS-PAGE and Western Blot

QS-21 was administered i.m. to mice in both hind legs and the iliac lymph nodes (ILN) were recovered at 1, 3 or 6 hours. The ILNs were rinsed twice in ice cold PBS and transferred into a pre-chilled glass homogenisation tube. 200 μl of RIPA buffer (PBS with 1% NP-40, 0.5% Na deoxycholate and 0.1% SDS) containing protease (cOmplete Mini Protease Inhibitor Cocktail Tablet, Roche) and phosphatase inhibitors (PhosSTOP, Roche) was transferred into the tube and the tissue was homogenised with a pestle. The homogenate was incubated on ice for 20 minutes and cleared by centrifugation at 12000 g for 20 minutes at 4 °C. Protein concentration was measured with the Micro BCA Protein Assay kit (Pierce) and 20 μg of protein was loaded onto a 12% Bis-Tris polyacrylamide gel. Gels were run in NuPAGE MOPS SDS Running Buffer (Invitrogen) at 150 V for 1 hour. Proteins were transferred onto a PVDF membrane (Amersham) for 1 h at 100 V, blocked in TBS-Tween containing 5% BSA and detected with anti-Caspase-1 p10 (Santa Cruz sc-515), anti- IL-1β (Cell Signaling #2021) and anti-Actin (Sigma) antibodies following manufacturer’s instructions.

### IL-1β ELISA

Bone marrow-derived dendritic cells from WT, Caspase-1 KO or NALP3 KO mice were differentiated as previously described^44^. The cells were stimulated with MPL (10 μg/ml – GSK), Alum (250 μg/ml), liposomal QS-21 (10 μg/ml) or a combination of MPL + Alum or MPL + QS-21 and IL-1β was detected in the supernatant by ELISA (R&D Systems) following manufacturer’s instructions.

### QS-21 labelling and immunofluorescence

QS-21-BODIPY (formulated in AS01) was produced as reported in ref. 6. Frozen cryostat sections of draining lymph nodes (DLN) (5 mm) from mice immunised with QS-21- BODIPY were fixed in acetone. Ab staining of DLN sections (CD11b, CD169 – clone MOMA-1- and B cells, biotinylated rat anti-mouse CD45R/B220; BD Biosciences) was performed in PBS/2% donkey serum, overnight at 4 °C, in conjunction with Dylight594-conjugated streptavidin (for CD45R/ B220). Tissue sections were examined with a confocal laser scanning microscope (Zeiss LSM 780 Meta). Minor brightness and contrast adjustments were made using Zeiss and/or other routine image-manipulation software and were applied uniformly to the whole image.

### Innate cell phenotyping in muscle and lymph node

Pooled tissues (gastrocnemius muscle or iliac lymph node [ILN]) from three immunised mice were treated by mechanical dissociation in 3 ml DMEM containing DNase I (100 mg/ml; Roche), 1% FCS and Liberase (Roche) at 0.1 U/ml (muscle), or 0.26 U/ml (ILN) for 30 min under agitation at 37 °C (muscle) or at room temperature (ILN). Liberase digestion was stopped by adding 10 mM EDTA and incubating on ice. Larger clumps of material were removed by passing the preparation through a 100 mM nylon cell strainer (BD Biosciences). The muscle preparation was enriched for hematopoietic cells by centrifugation on a Percoll gradient (Amersham-Pharmacia). Cells were washed twice and resuspended in PBS containing 2 mM EDTA and 2% FCS. After treatment with 2.4G2 Ab for 5 min to block the FcR, the cells were stained with the following anti-mouse Abs: anti–Ly6C-FITC, the PE- conjugated lineage identifiers (anti-SiglecF, anti-CD8a, anti-CD4, anti- CD19, and anti-NK1.1), anti–Ly6G-PerCP, anti–Ly6C-PerCP, anti–MHC class II (MHCII) (I-A/I-E)-Alexa700, anti–CD11b-PB, anti–CD11c-PECy7, anti-CD169 (clone MOMA-1) APC-Cy7–conjugated lineage identifiers (anti-CD3, anti-CD19, and anti-Ly6G), and anti–CD45-PO. All Abs were obtained from BD Biosciences, except the anti-CD169 that was from BMA Biomedicals. Fluorescent events were acquired using an LSR2 and analysed using FACSDiva software (BD Biosciences). Monocytes were defined as Ly6C high CD11b^+^ Ly6G- cells, and neutrophils were defined as SSC high CD11b^+^Ly6G/ Gr1^high^. After exclusion of neutrophil, monocyte and lymphocyte populations, DCs were gated as CD11c + MHCII+ from muscles or as CD11c mid MHCII high from ILNs.

### Statistical analyses

Statistical analyses were performed with GraphPad Prism software. Statistical significance was determined with Mann-Whitney non-parametric tests. p ≥ 0.05: not significant; p < 0.05 and ≥0.01: *p < 0.01 and ≥0.001: **p < 0.001 and ≥0.0001: ***p < 0.0001: ****.

## Additional Information

**How to cite this article**: Detienne, S. *et al*. Central Role of CD169^+^ Lymph Node Resident Macrophages in the Adjuvanticity of the QS-21 Component of AS01. *Sci. Rep.* **6**, 39475; doi: 10.1038/srep39475 (2016).

**Publisher's note:** Springer Nature remains neutral with regard to jurisdictional claims in published maps and institutional affiliations.

## Supplementary Material

Supplementary Information
