# Supplementary material for: Central Role of CD169+ Lymph Node Resident Macrophages in the Adjuvanticity of the QS-21 Component of AS01
Source: Sci Rep. 2016 Dec 20;6:39475. doi: 10.1038/srep39475 (PMC5172233; doi:10.1038/srep39475)
Supplement: Supplementary Information [file srep39475-s1.pdf]

Central Role of CD169$^+$ Lymph Node Resident Macrophages in the Adjuvanticity of the QS-21 Component of AS01.

Sophie Detienne[1*], Iain Welsby[1*], Catherine Collignon[2], Sandrine Wouters[2], Margherita Coccia[2], Sophie Delhaye[2], Laurye Van Maele[1], Séverine Thomas[1], Maëlle Swertvaegher[1], Aurélie Detavernier[1], Abdelatif Elouahabi[2], Stanislas Goriely[1†] and Arnaud Didierlaurent[2†].

[1]Institute for Medical Immunology (IMI), Université Libre de Bruxelles, Rue Adrienne Bolland 8, B-6041 Gosselies, Belgium.

[2]GSK Vaccines, Rue de l'Institut 89, B-1330 Rixensart, Belgium

[*] and [†]: These authors contributed equally to the work.

Supplementary Figures

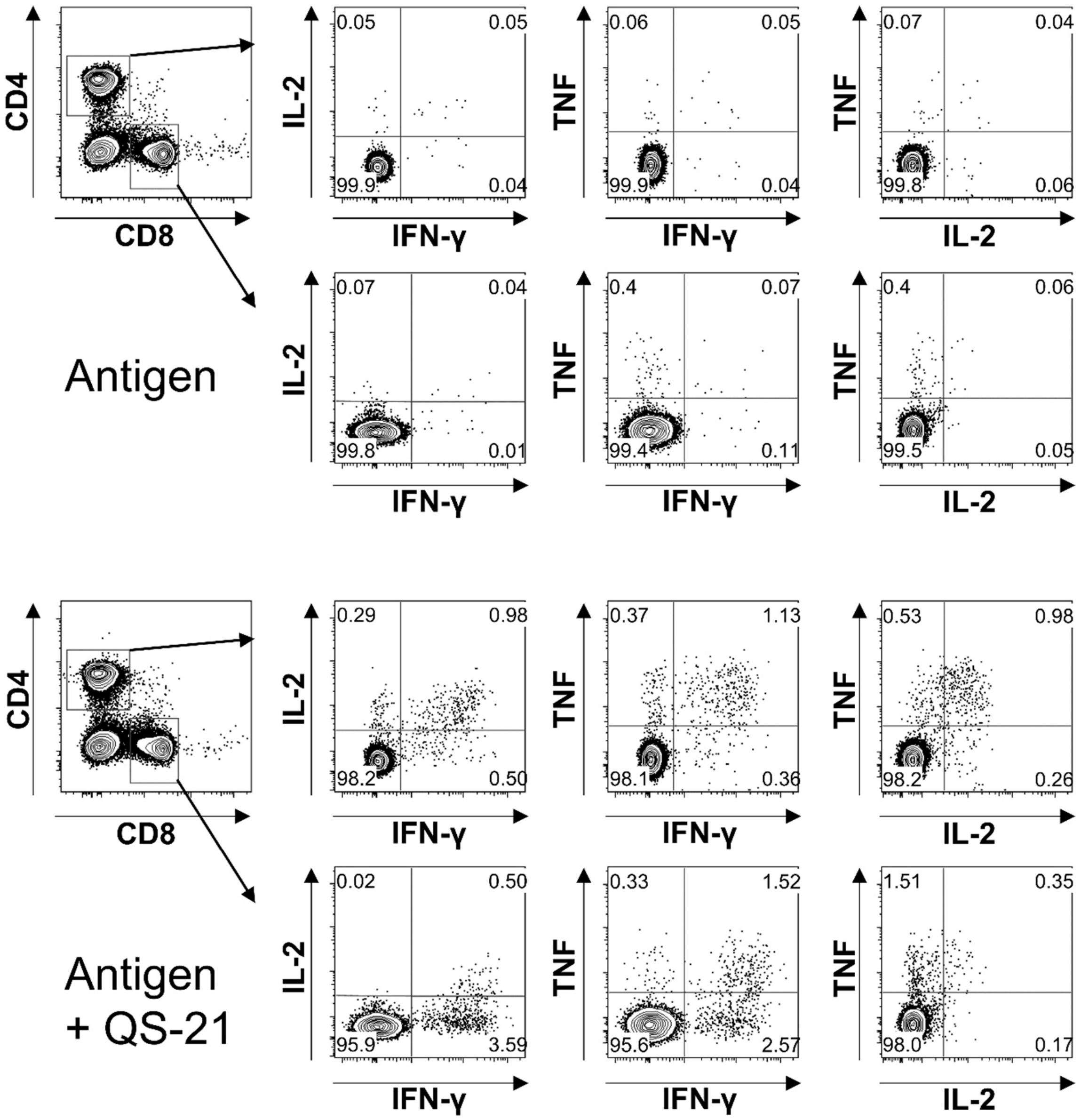

Supplementary Figure 1. Intracellular cytokine stain. Following antigenic peptide restimulation, T cells were gated on either CD4 or CD8-positive cells and IL-2, IFN-γ or TNF production was assessed. One mouse immunised with antigen alone and one mouse immunised with QS-21-adjuvanted antigen are shown.

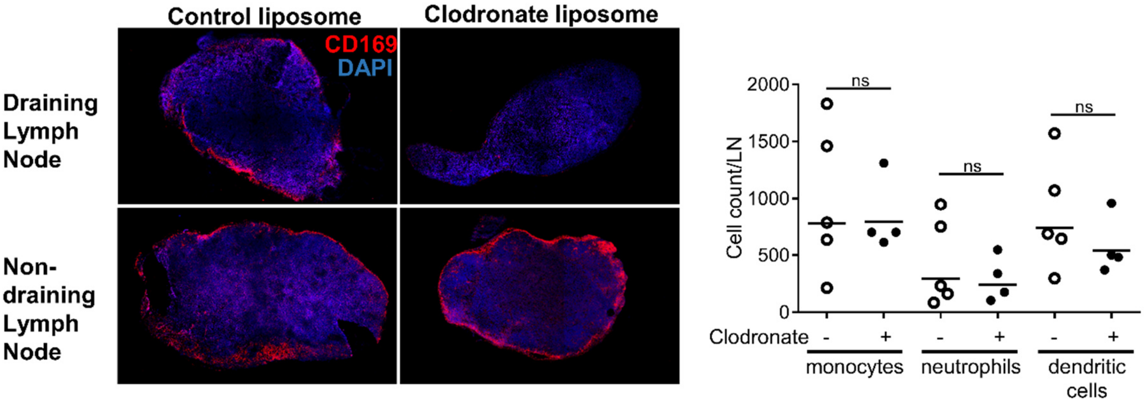

Supplementary Figure 2. Related to figure 4. A) 6 days after clodronate liposome injection, the draining (iliac) and non-draining (cervical) lymph nodes were recovered and stained with DAPI (nuclei - blue) and anti-CD169 (red) antibodies. B) DLNs were recovered at day 6 post clodronate liposome (CL) injection. Monocytes ($Lin^{-}CD11b^{+}$ $Ly6C^{+}$ $Ly6G^{-}$), neutrophils (SSC^{hi} Lin^{-} $CD11b^{+}$ $Ly6C^{int}$ $Ly6G^{+}$) and dendritic cells ($Lin^{-}CD11c^{+}$ IA/IE^{hi}) were quantified by flow cytometry (n=4-5).

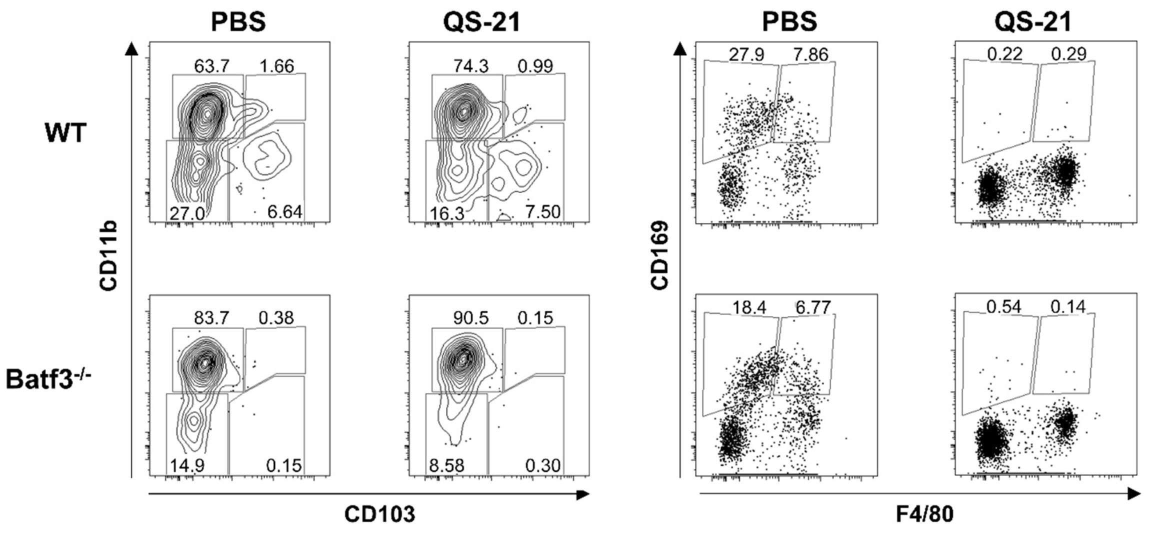

Supplementary Figure 3. Related to figure 5. 24h after PBS or QS-21 injection, dendritic cell and CD169+ cell subset frequencies in WT and Batf3$^{-/-}$ mice were analysed by flow cytometry.

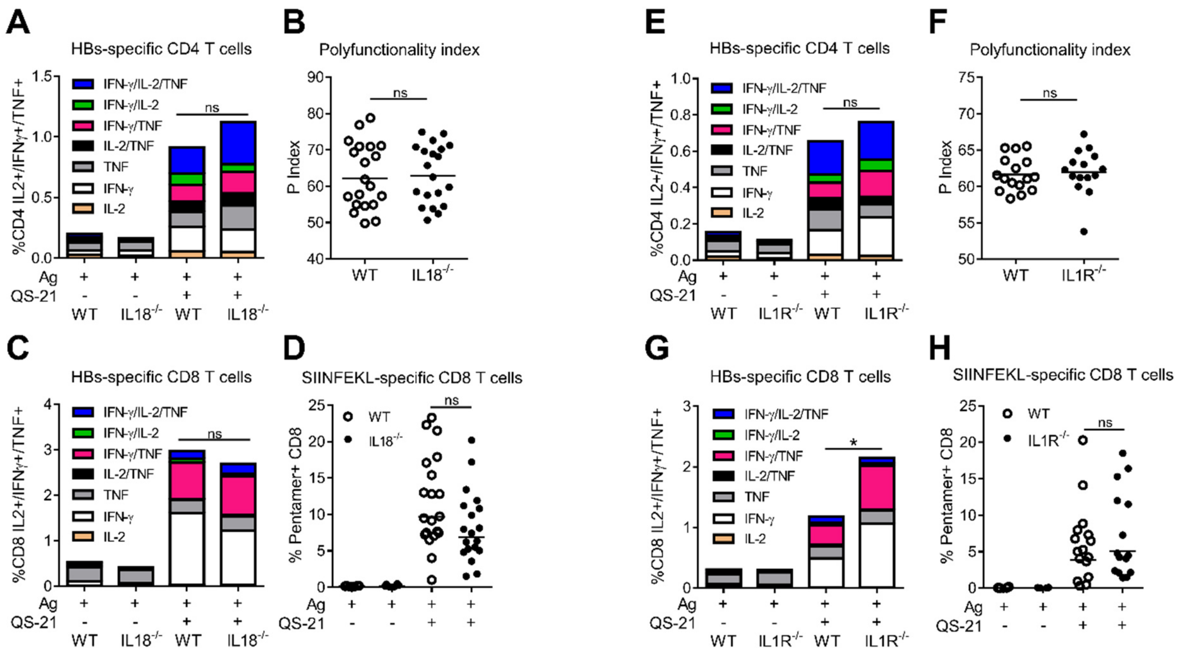

Supplementary Figure 4. *Antigen-specific T cell responses are normal in IL-18 and IL-1R KO mice.* A-B) WT and IL-18 KO mice were immunised as in figure 1A. At day 21, cytokine production (IL-2, IFN-γ, TNF) of HBs-specific CD4 (A) and CD8 (C) T cells were evaluated by intracellular staining. The data is represented as median of 6 (Ag) or 20 (QS-21) mice. CD4 T cell polyfunctionality index was calculated as described (B) and the frequency of OVA-specific circulating CD8 T-cells (D) was assessed by pentamer staining (Ag: n=6, QS-21: n=20). WT and IL-1 receptor KO mice were immunised as in 1A. At day 21, cytokine production (IL-2, IFN-γ, TNF) of HBs-specific CD4 (E) and CD8 (G) T cells were evaluated by intracellular staining. The data is represented as the median value of 4 (Ag) or 16 (QS-21) mice. The CD4 T cell polyfunctionality index was calculated as described (F) and the frequency of OVA-specific circulating CD8 T-cells (H) were assessed by pentamer staining (Ag: n=6, QS-21: n=16)

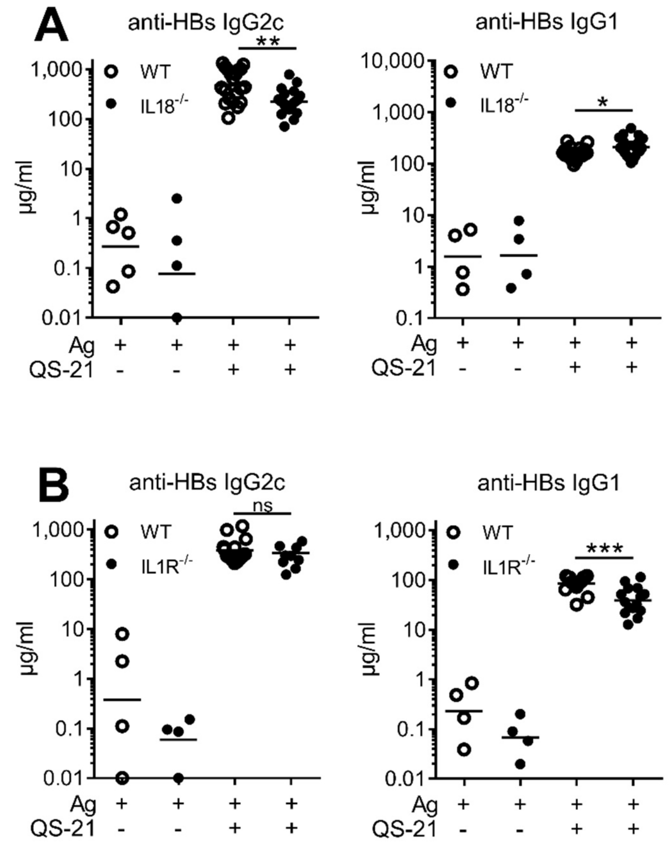

Supplementary Figure 5. *Antibody production partially depends on IL-18 and IL-1R-mediated signalling events.* WT and IL-18 KO (A) or IL1-R KO (B) mice were immunised as in 1A. Anti-HBs IgG1 and IgG2c in the serum at day 21 were measured by ELISA (Ag: n=4-5, QS-21: n=20). Each point represents a single mouse and the horizontal bar represents the geometric mean. Statistical significance was determined by a non-parametric Mann Whitney t-test. The data represents a pool of 2 independent experiments.

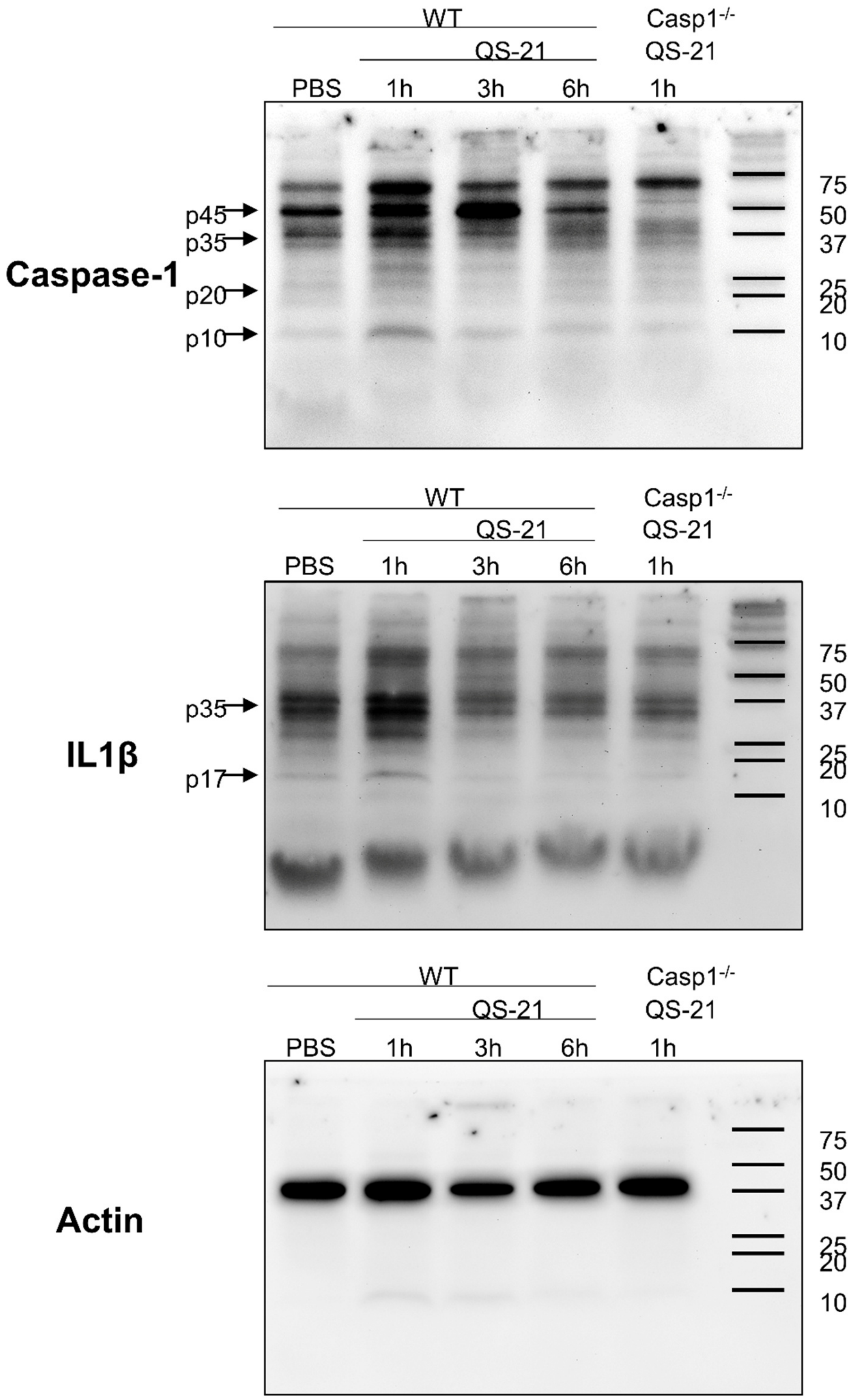

Supplementary Figure 6. Uncropped immunoblots for Figure 6B
